# Evaluation of assays for thyroxine, triodotyronine and thyroid-stimulating hormone on the ARIA II automated radioimmunoassay instrument

**DOI:** 10.1155/S1463924683000139

**Published:** 1983

**Authors:** M. J. Duffy, J. Tuttlebee, F. O'Sullivan, M. Allen-Davis

**Affiliations:** Department of Nuclear Medicine St. Vincent's Hospital Dublin 4 Ireland


Journal of Automatic Chemistry, Volume 5, Number 1 (January–March 1983), pages 43–44

### Evaluation of assays for thyroxine, triodotyronine and thyroid-stimulating hormone on the ARIA II automated radioimmunoassay instrument

**M. J. Duffy, J. Tuttlebee, F. O'Sullivan and M. Allen-Davis**

*Department of Nuclear Medicine, St. Vincent's Hospital, Dublin 4, Eire*

### Introduction

One of the most significant advances in clinical chemistry over the past 15 years has been the automation of many commonly requested tests. Automation has allowed the performance of many more assays than could be accomplished by manual methods and, at the same time, has led to increased precision.

One area of clinical chemistry where automation has had relatively little impact is radioimmunoassay, which because of the multiple pipetting of reagents is very labour-intensive and yields results with relatively poor precision. Automation of these tests would thus offer distinct advantages over existing manual procedures.

The main problem in the automation of radioimmunoassay has been the separation of free from antibody-bound antigen. Over the past few years this problem has been overcome, and today at least eight different automated radioimmunoassay systems are in use around the world (for a review see reference [1]). In this paper an evaluation of one of these instruments is described: the ARIA II for the assay of thyroxine (T4), triodotyronine (T3) and thyroid-stimulating hormone (TSH).

### Materials and methods

The ARIA II instrument and the reagents for the automated analysis of T4, T3 and TSH were purchased from Becton Dickinson Immunodiagnostics, Salt Lake City, Utah, USA. This instrument utilizes a continuous-flow system, with separation of free and antibody-bound antigen occurring in antibody chambers. In these chambers antibody is covalently bound to solid supports. This type of separation system has a number of advantages, which include economy, as the same antibody chamber is used over and over; precision—exactly the same amount of antibody reacts in each assay; and rapidity, since the physical structure provides a large number of reactive antibody sites and thus makes reaction time very short (2 min for some assays).

Manual kits were obtained from the following sources: T4 (Amerlex) from Amersham, Buckinghamshire, UK; T3 (Dac-Cel) from Wellcome Reagents Ltd, London; and TSH from Corning Medical and Scientific, Halstead, Essex, UK. The separation system used in all of these assays is based on the attachment of antibody to a solid support. Lyphochek TM trilevel quality-control sera was supplied by Bio-Rad Laboratories Ltd, Watford, Hertfordshire, UK.

Within-assay variation was determined using the quality-control sera randomly distributed amongst the patients samples. Between-assay variation was calculated using the same pool of control sera spread over at least nine consecutive assays. Carry-over was measured by the method of Broughton *et al.* [2]. At least a six-fold concentration difference of analyte was used in the sera for investigating carry-over.

### Results

The within-assay and between-assay variation for T4, T3 or TSH at three different concentration levels is shown in table 1. The correlations between T4, T3 and TSH and respective manual methods are shown in figures 1, 2 and 3. High-to-low and low-to-high interaction for the three hormones is shown in table 2.

### Discussion

In general, the precision obtained for T4, T3 and TSH in the present investigation compares favourably with that obtained with manual kits. The only exception was the relatively poor coefficient of variation for low levels of TSH. Our data on precision for T4 and T3 is similar to that obtained by Chen *et al.* [3] who also used the ARIA. Similar precision for T4 has also been reported using the Kemtek 3000 automated immunoassay system [4]. Less good precision, however, has been reported for T4 and T3 on the Micromedic automated system [4]. TSH assays do not appear to have been evaluated on any automated instrument to date.

In this investigation, good correlations with little bias were found between results obtained on the automated system and those from manual methods. Similar good agreement between automated methods and manual methods for T4 and T3 have been previously reported by Chen *et al.* [3]. However, in contrast to the findings by these workers, no significant problem with carry-over was encountered.

*Table 1. Reproducibility of assays for T4, T3 and TSH using the ARIA II system. Number of determinations shown in parenthesis.*

| | Within-assay variation | | | Between-assay variation | | |
|---|---|---|---|---|---|---|
| Assay | Mean | CV | *N* | Mean | CV | *N* |
| (T4 n mol/l) | 23·31 | 6·44 | (9) | 27·86 | 7·57 | (14) |
| | 75·29 | 2·58 | (10) | 75·39 | 7·30 | (12) |
| | 167·3 | 3·08 | (10) | 168·4 | 4·60 | (13) |
| (T3 n mol/l) | 1·58 | 10·71 | (13) | 1·80 | 11·75 | (12) |
| | 3·31 | 3·27 | (11) | 4·09 | 7·31 | (12) |
| | 7·20 | 6·40 | (13) | 7·30 | 7·52 | (12) |
| TSH (mU/l) | 4·0 | 15·3 | (10) | 4·4 | 14·7 | (9) |
| | 24·0 | 8·0 | (10) | 25·8 | 8·64 | (9) |

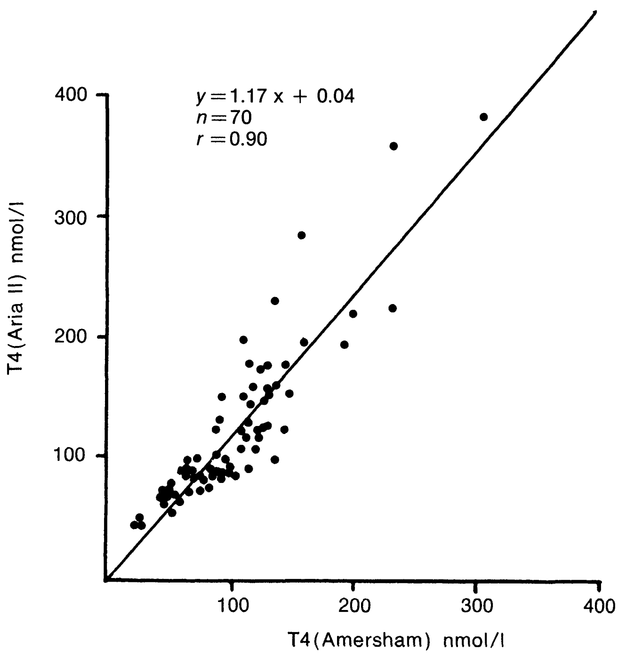

*Figure 1. Correlation between T4 values determined by an automated (ARIA II) method and a manual method (Amerlex Kit, Amersham).*

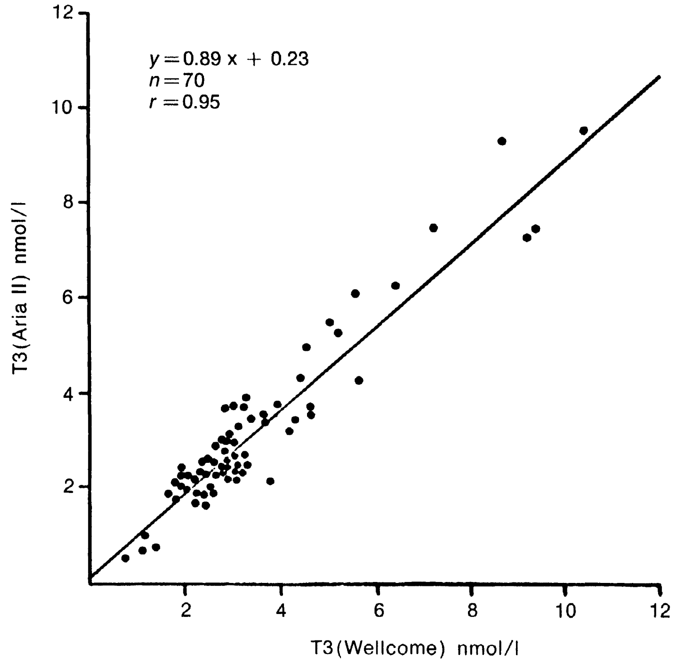

*Figure 2. Correlation between T3 values determined by an automated (ARIA II) method and a manual method (Dac-Cel, Wellcome).*

*Table 2. Carry-over on assays for T4, T3 and TSH using the ARIA II system. Each determination was carried out six times.*

| | High to low (%) | Low to high (%) |
|---|---|---|
| T4 | 2·12 | 4·4 |
| T3 | 2·74 | 4·1 |
| TSH | 1·15 | 5·6 |

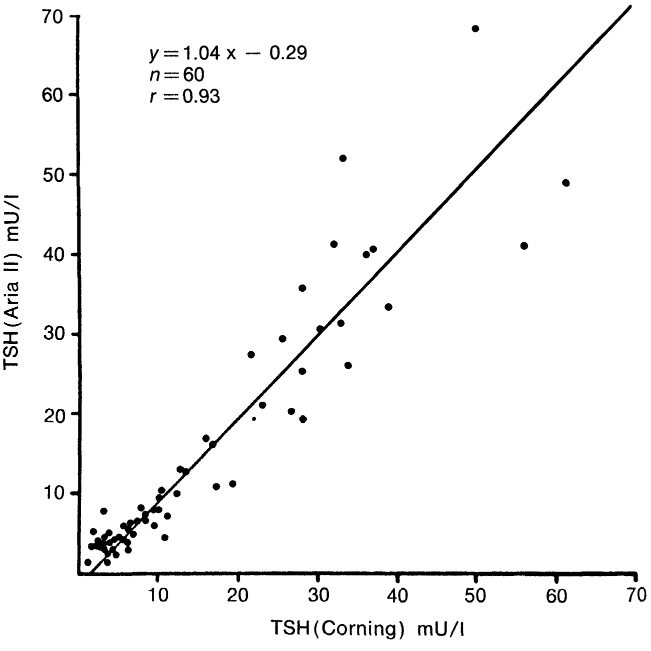

*Figure 3. Correlation between TSH values determined by an automated (ARIA II) method and a manual method (Corning).*

*Table 3. Times (in min) of different assay parameters for T4, T3 and TSH on the ARIA II system, one cycle is equivalent to one tube in a manual radioimmunoassay.*

| Parameter | T4 | T3 | TSH |
|---|---|---|---|
| Incubation | 0 | 20 | 120 |
| Throughtime | 2·2 | 22 | 125 |
| Interval between results print-out | 2·2 | 2 | 5 |
| Assay time for 60 cycles | 132 | 140 | 503 |

In conclusion, the ARIA II system has a useful role in a medium-sized radioimmunoassay laboratory. It is almost a hands-off instrument and yields results with acceptable precision. Its main disadvantage is that it is relatively slow in generating results, especially for TSH (see table 3). However, the multichannel systems presently being developed should increase throughput.

### References

1. Forrest, G. C., *Immunoassay for Clinical Chemistry*, Ed. Hunter, W. M. and Corrie, J. E. T. (Churchill Livingstone, Edinburgh, 1983), 211.
2. Broughton, P. M. G., Gowenlock, A. H., McCormack, J. J. and Neill, D. W., *Annals of Clinical Biochemistry*, **11** (1974), 207.
3. Chen, I. W., Maxon, H. R., Heminger, L. A., Ellis, K. S. and Volle, C. P., *Journal of Nuclear Medicine*, **21** (1980), 1162.
4. Kadury, S., John, R., Woodhead, J. S. and Kurtz, A. B., *Annals of Clinical Biochemistry*, **18** (1981), 97.

